# Supplementary material for: The cortical microenvironment drives early immune organization and controls early osteoclastogenesis in bone healing
Source: Nat Commun. 2026 Aug 27;17:9154. doi: 10.1038/s41467-026-77196-7 (PMC13522349; doi:10.1038/s41467-026-77196-7)
Supplement: Supplementary file 1 [file 41467_2026_77196_MOESM10_ESM.pdf]

nature portfolio

Corresponding author(s): Georg N. Duda

Last updated by author(s): Jul 22, 2026

Reporting Summary

Nature Portfolio wishes to improve the reproducibility of the work that we publish. This form provides structure for consistency and transparency in reporting. For further information on Nature Portfolio policies, see our Editorial Policies and the Editorial Policy Checklist.

Statistics

For all statistical analyses, confirm that the following items are present in the figure legend, table legend, main text, or Methods section.

n/a	Confirmed	
☐	☒	The exact sample size (*n*) for each experimental group/condition, given as a discrete number and unit of measurement
☐	☒	A statement on whether measurements were taken from distinct samples or whether the same sample was measured repeatedly
☐	☒	The statistical test(s) used AND whether they are one- or two-sided<br>*Only common tests should be described solely by name; describe more complex techniques in the Methods section.*
☒	☐	A description of all covariates tested
☐	☒	A description of any assumptions or corrections, such as tests of normality and adjustment for multiple comparisons
☐	☒	A full description of the statistical parameters including central tendency (e.g. means) or other basic estimates (e.g. regression coefficient) AND variation (e.g. standard deviation) or associated estimates of uncertainty (e.g. confidence intervals)
☐	☒	For null hypothesis testing, the test statistic (e.g. *F*, *t*, *r*) with confidence intervals, effect sizes, degrees of freedom and *P* value noted<br>*Give P values as exact values whenever suitable.*
☒	☐	For Bayesian analysis, information on the choice of priors and Markov chain Monte Carlo settings
☐	☒	For hierarchical and complex designs, identification of the appropriate level for tests and full reporting of outcomes
☒	☐	Estimates of effect sizes (e.g. Cohen's *d*, Pearson's *r*), indicating how they were calculated

Our web collection on statistics for biologists contains articles on many of the points above.

Software and code

Policy information about availability of computer code

Data collection	Single-cell RNA sequencing data were generated using the Chromium Controller (10x Genomics, Single Cell 3′ v3 workflow) and sequenced on an Illumina NextSeq 500 using vendor-provided acquisition software. Histological and TRAP-stained images were acquired using a Leica DM6B microscope with LAS X Office software. MELC imaging data were collected using BioDecipher® or Toponome Image Cycler® systems with MelTec TIC-Control software. No custom software was used for data collection.
Data analysis	scRNAseq data were processed using Cellranger count (v7.1.0) (10X Genomics). Image analysis and cell segmentation were performed using CellProfiler (v.4.2.8). All downstream analyses, both scRNAseq and histological analyses, were performed using R software (v.4.3.3). R packages included Seurat (v5.1.0), Harmony (v1.2.3), DittoSeq (v1.14.3) and SPIAT (v1.0.0)

For manuscripts utilizing custom algorithms or software that are central to the research but not yet described in published literature, software must be made available to editors and reviewers. We strongly encourage code deposition in a community repository (e.g. GitHub). See the Nature Portfolio guidelines for submitting code & software for further information.

Data

Policy information about availability of data

All manuscripts must include a data availability statement. This statement should provide the following information, where applicable:

- Accession codes, unique identifiers, or web links for publicly available datasets
- A description of any restrictions on data availability
- For clinical datasets or third party data, please ensure that the statement adheres to our policy

Single-cell RNA-seq datasets (raw FASTQ files and processed count matrices) generated in this study are available at the Gene Expression Omnibus under accession number GSE305266. All Seurat objects as well as all MELC data are available in the Zenodo open-access repository (https://zenodo.org/): the Seurat objects are deposited under https://doi.org/10.5281/zenodo.16871641, the MELC images of intact controls can be found under https://doi.org/10.5281/zenodo.16870991, the MELC image of young animals at day 3 under https://doi.org/10.5281/zenodo.16870196, and the MELC images of young animals at day 7 under https://doi.org/10.5281/zenodo.16871121. Finally, the CellProfiler pipeline and its respective output files can be found under https://doi.org/10.5281/zenodo.16876039. All Zenodo records are interlinked within the public repository and can be accessed directly via their respective DOIs. Additionally, source data are provided with this paper.

Research involving human participants, their data, or biological material

Policy information about studies with human participants or human data. See also policy information about sex, gender (identity/presentation), and sexual orientation and race, ethnicity and racism.

Reporting on sex and gender	NA
Reporting on race, ethnicity, or other socially relevant groupings	NA
Population characteristics	NA
Recruitment	NA
Ethics oversight	NA

Note that full information on the approval of the study protocol must also be provided in the manuscript.

Field-specific reporting

Please select the one below that is the best fit for your research. If you are not sure, read the appropriate sections before making your selection.

[x] Life sciences [ ] Behavioural & social sciences [ ] Ecological, evolutionary & environmental sciences

For a reference copy of the document with all sections, see nature.com/documents/nr-reporting-summary-flat.pdf

Life sciences study design

All studies must disclose on these points even when the disclosure is negative.

Sample size	Sample sizes were chosen based on previous studies using comparable fracture healing models and single-cell transcriptomic approaches, as well as practical considerations related to animal availability and experimental complexity. No formal statistical methods were used to predetermine sample size. The selected sample sizes were sufficient to reproducibly detect robust differences in cellular composition, transcriptional states and spatial organization between experimental conditions.
Data exclusions	No data was excluded
Replication	For scRNAseq experiments, samples were generated by pooling tissue from 4 animals per biological sample, and analyses were performed across 4-5 replicates per condition. Histological analyses were conducted on independent animals and yielded comparable spatial patterns and trends for 5-6 replicates. No data were excluded on the basis of reproducibility.
Randomization	Age groups were predefined in the experimental design. Within each age group, animals were randomly assigned to fracture or control conditions and to the respective post-injury timepoints.
Blinding	Blinding was not applied during animal allocation or surgical procedures because fracture induction and timepoint assignment are inherent to the experimental design and cannot be concealed from the operator. Downstream analyses were performed using automated or predefined computational pipelines with objective outcome measures, minimizing the potential for observer bias. No subjective outcome scoring was used to determine primary results.

Reporting for specific materials, systems and methods

We require information from authors about some types of materials, experimental systems and methods used in many studies. Here, indicate whether each material, system or method listed is relevant to your study. If you are not sure if a list item applies to your research, read the appropriate section before selecting a response.

Materials & experimental systems

n/a	Involved in the study
☐	☒ Antibodies
☒	☐ Eukaryotic cell lines
☒	☐ Palaeontology and archaeology
☐	☒ Animals and other organisms
☒	☐ Clinical data
☒	☐ Dual use research of concern
☒	☐ Plants

Methods

n/a	Involved in the study
☒	☐ ChIP-seq
☐	☒ Flow cytometry
☒	☐ MRI-based neuroimaging

Antibodies

Antibodies used	The following antibodies were used in the manuscript: CD45-BV711 (BD Horizon, Cat# 563709, clone 30-F11, lot 3072697, dilution 1:400); Ly6G-FITC (BioLegend, Cat# 127606, clone 1A8, lot B435608, dilution 1:200); Ly6C-PerCP-eFluor710 (Invitrogen, Cat# 45-5932-92, clone HK1.4, lot 1996378, dilution 1:400); CD163-PE (Invitrogen, Cat# 12-163-182, clone TKNUPJ, lot 2896790, dilution 1:100); CD14-PE-Dazzle (BioLegend, Cat# 123326, clone Sa14-2, lot B398450, dilution 1:100); NK1.1-PE-Cy7 (Invitrogen, Cat# 25-5941-82, clone PK136, lot 2686516, dilution 1:100); F4/80-eFluor660 (Invitrogen, Cat# 50-4801-82, clone BM8, lot 2989386, dilution 1:100); CD11b-APC-eFluor780 (Invitrogen, Cat# 47-0112-82, clone M1/70, lot 2560618, dilution 1:150); CD3e-biotin (Invitrogen, Cat# 13-0031-82, clone 145-2C11, lot 3053659, dilution 1:300); CD5-biotin (Invitrogen, Cat# 13-0051-85, clone 53-7.3, lot 2355944, dilution 1:150); CD19-biotin (Invitrogen, Cat# 13-0191-85, clone MB19.1, lot 2433463, dilution 1:150); NKp46-biotin (Invitrogen, Cat# 13-3351-82, clone 29A1.4, lot 2518332, dilution 1:100); Siglec-F-biotin (BioLegend, Cat# 155512, clone S17007L, lot B488098, dilution 1:150); Streptavidin V500 (BD Horizon, Cat# 561419, lot 4015931, dilution 1:800).
Validation	All antibodies used in this study were commercially obtained and validated by the manufacturers for the indicated applications. Antibody specificity was further confirmed through the use of established positive and negative control populations, expected staining patterns, and consistency with previously published studies.

Animals and other research organisms

Policy information about studies involving animals; ARRIVE guidelines recommended for reporting animal research, and Sex and Gender in Research

Laboratory animals	C57BL/6NRj mice, 12-week-old and 52-week-old; Tnf ΔARE/+ mice were generated and kindly provided by George Kollias
Wild animals	This study did not involve wild animals.
Reporting on sex	This study was conducted exclusively in female mice. Female mice were chosen because bone healing is delayed relative to males, allowing improved resolution of early immune and osteoclastogenic processes. to minimize biological variability, sex was not included as an experimental variable.
Field-collected samples	This study did not involve samples collected from the field.
Ethics oversight	All experiments were carried out with ethical permission according to the policies and principles established by the Animal Welfare Act, the National Institutes of Health Guide for Care and Use of Laboratory Animals, and the National Animal Welfare Guidelines, the ARRIVE guidelines and were approved by the local legal representative animal rights protection authorities (G0012/22; Landesamt für Gesundheit und Soziales Berlin).

Note that full information on the approval of the study protocol must also be provided in the manuscript.

Plants

Seed stocks	NA
Novel plant genotypes	NA
Authentication	NA

Flow Cytometry

Plots

Confirm that:

- [x] The axis labels state the marker and fluorochrome used (e.g. CD4-FITC).
- [x] The axis scales are clearly visible. Include numbers along axes only for bottom left plot of group (a 'group' is an analysis of identical markers).
- [x] All plots are contour plots with outliers or pseudocolor plots.
- [x] A numerical value for number of cells or percentage (with statistics) is provided.

Methodology

Sample preparation	Cells were stained with a mixture of lineage markers (biotinylated CD3e, CD5, CD19, NKp46) marked by streptavidin in BV500, CD45-BV711, Ly6G-FITC, Ly6C-PerCP-eF710, CD163-PE, CD14-PE-Dazzle, NK1.1-PE-Cy7, F4/80- eFluor660, and CD11b-APC-eFluor780. Dead cells were excluded based on Fixable Viability Dye (FVD-V450) incorporation and cells of interest were isolated from the CD45+Ly6G-NK1.1- fraction as CD163hiF4/80hi for tissue resident macrophages and CD14+F4/80lo for putative Spp1hi cells in a Sony MA90 sorter.
Instrument	Sony MA90 sorter, MacsQuant10 cytometer
Software	FlowJo
Cell population abundance	Cell numbers in each of the sorted fractions were quantified as FVD-V450-, in a 1:100 dilution in PBS, in a MacsQuant10 cytometer. Between 30000-70000 cells were obtained per population.
Gating strategy	Intact cells were gated through FSC-A and SSC-A. Doublets were excluded according to FSC-A and FSC-H and Fixable Viability Dye incorporation was used to discriminate against non-viable cells. Gating strategy for the populations of interest is indicated in the methods.

- [x] Tick this box to confirm that a figure exemplifying the gating strategy is provided in the Supplementary Information.
