# Supplementary material for: The cortical microenvironment drives early immune organization and controls early osteoclastogenesis in bone healing
Source: Nat Commun. 2026 Aug 27;17:9154. doi: 10.1038/s41467-026-77196-7 (PMC13522349; doi:10.1038/s41467-026-77196-7)
Supplement: Supplementary file 2 [file 41467_2026_77196_MOESM1_ESM.pdf]

Supplementary information

Suppl. Figure 1

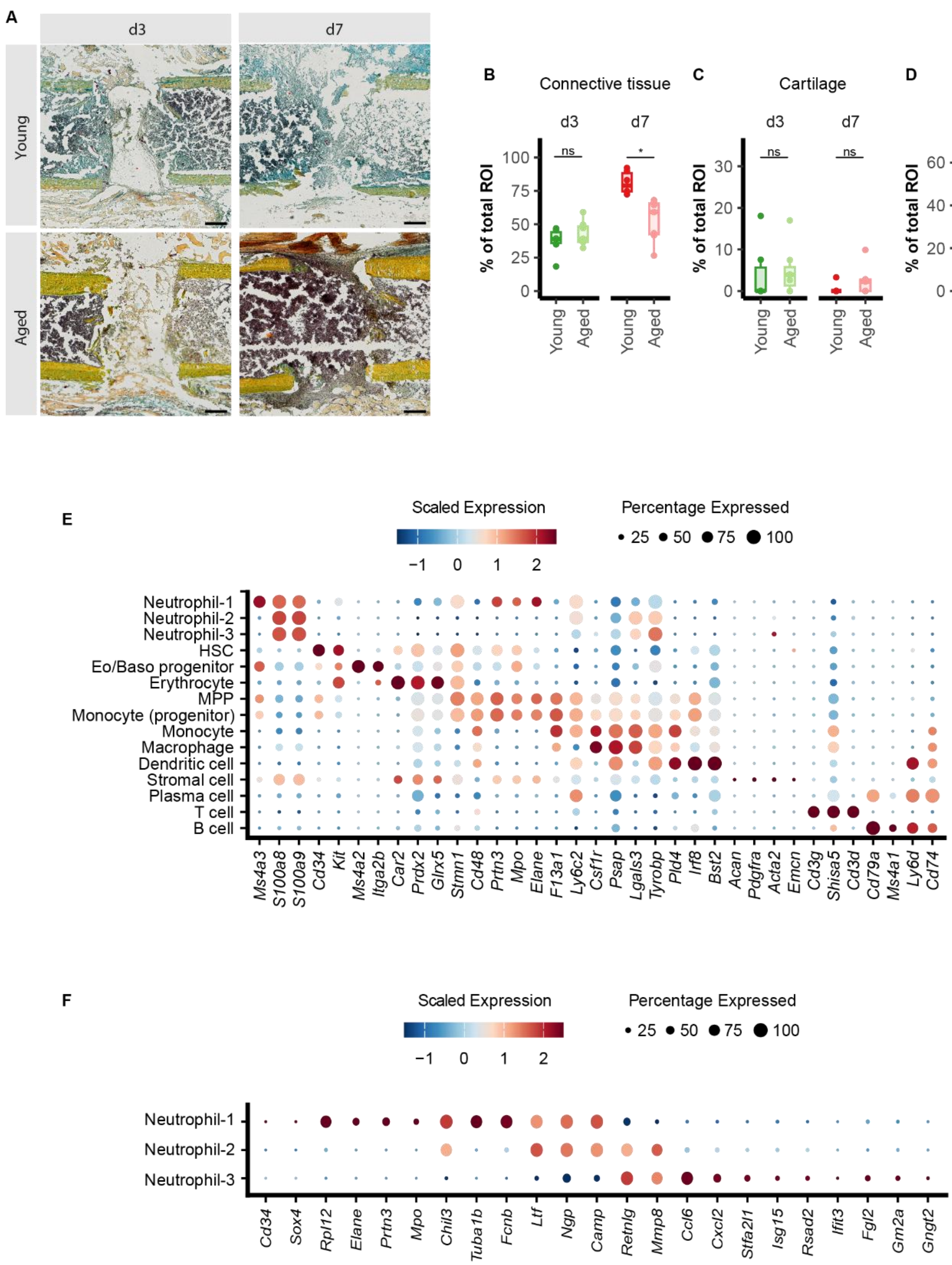

Suppl. Fig 1: Validation of the *in vivo* osteotomy model for early-phase fracture healing.

(A) Representative histological sections stained with Movat Pentachrome from young and aged mice at day 3 (d3) and day 7 (d7) post-osteotomy. Tissue components are visualized as follows: yellow = mineralized bone, red = connective tissue, and blue/green = cartilage. Sections are shown in standardized orientation (proximal to the left, distal to the right). Scale bar = 200 µm.

(B-D) Quantification of the relative area occupied by connective tissue (B), cartilage (C) and cancellous bone (D) within the defined region of interest (ROI = 0.7 mm fracture gap including 0.4 mm proximal and distal). Tissue segmentation was performed using a custom Fiji-based pipeline. Box plots display the median, interquartile range, and whiskers extending to the most extreme values within 1.5× the interquartile range. Individual data points represent biological replicates (Young d3, n = 6; Aged d3, n = 7; Young d7, n = 6; Aged d7, n = 6), and paired measurements from the same animal are connected by lines. Differences between cell populations were evaluated using paired one-sided Wilcoxon signed-rank tests; Connective tissue: d7 young-aged $P = 0.0022$; Cancellous bone: d3 young-aged $P = 0.0015$; ns = not significant.

(E-F) Dot plots displaying scaled expression and percentage of expressing cells for gene markers obtained from literature.

Data are provided in Supplementary Data 1.

Suppl. Figure 2

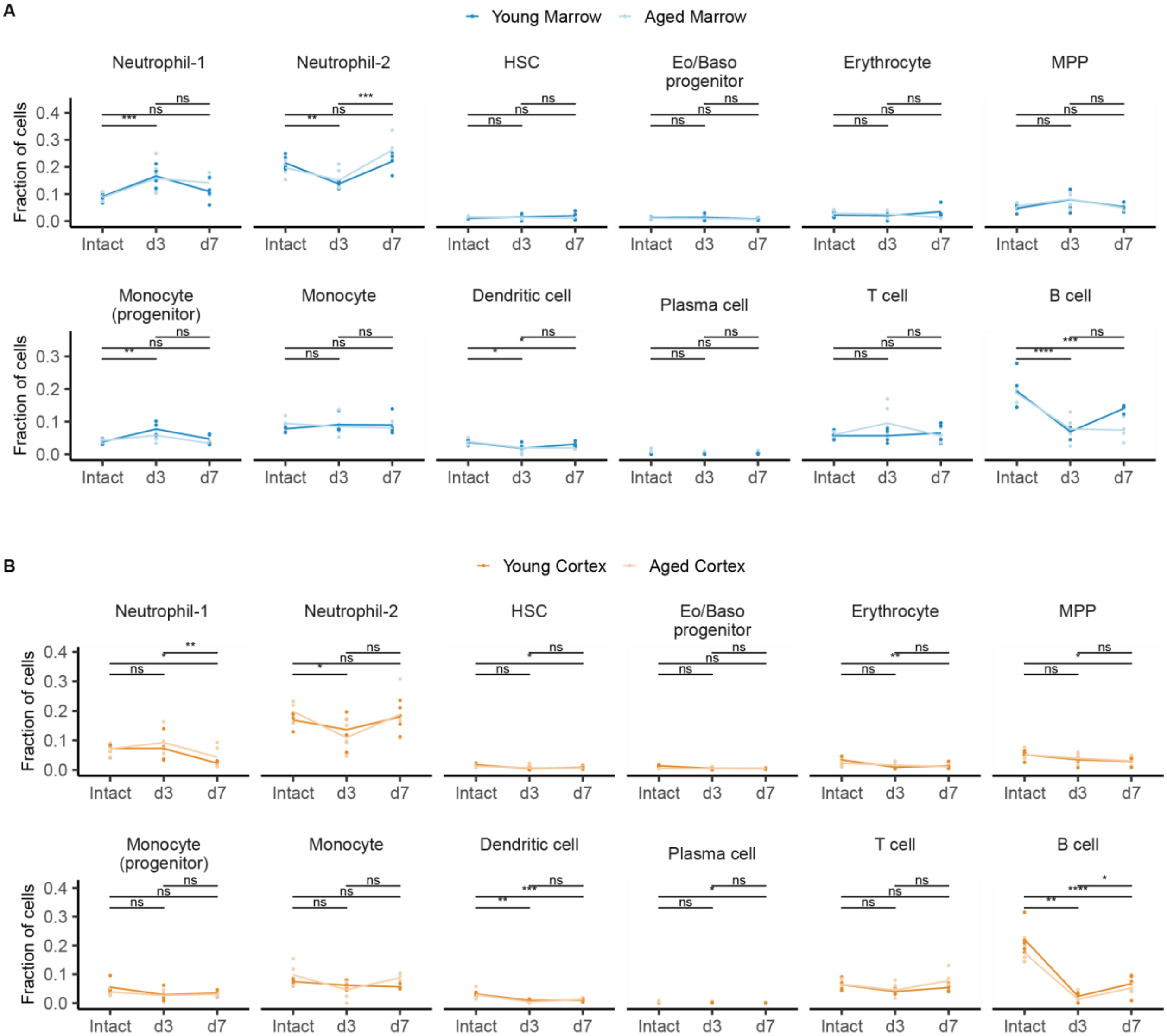

Suppl. Fig. 2: Temporal distribution of hematopoietic and immune populations during early fracture healing in marrow and cortex.

Fraction of cells per annotated population at baseline (intact) and post-osteotomy (day 3 and day 7) in marrow (A, blue) and cortex (B, orange). Cell types include Neutrophil-1, Neutrophil-2, hematopoietic stem cells (HSCs), eosinophil/basophil (Eo/Baso) progenitors, erythrocytes, multipotent progenitors (MPPs), monocyte progenitors, monocytes, dendritic cells, plasma cells and T and B cells. Each data point represents one biological replicate (Intact, n = 10; d3, n = 10; d7, n = 9). Lines connect age group means. Statistical comparisons were performed using two-sided Wilcoxon test with Benjamini-Hochberg correction and its *P* values can be found in the Suppl. Data file; * $P < 0.05$, ** $P < 0.01$, *** $P < 0.001$, **** $P < 0.0001$; ns = not significant.

Data are provided in Supplementary Data 2.

Suppl. Figure 3

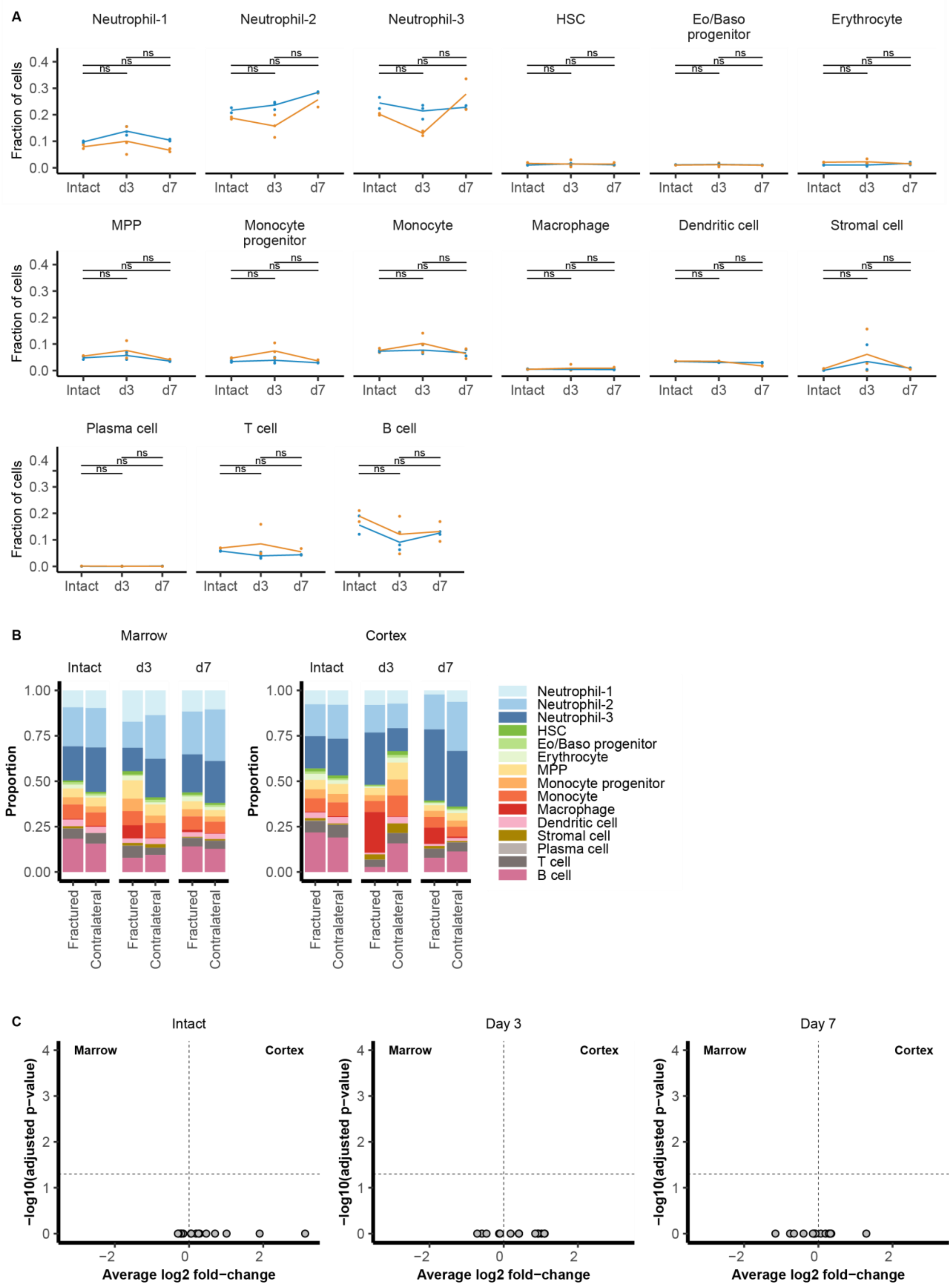

Suppl. Fig. 3: Stable baseline composition and compartmental consistency across timepoints and tissue locations.

(A) Proportional abundance of annotated populations across timepoints (intact, day 3 and day 7) in marrow (blue) and cortex (orange) of contralateral bones. Each data point represents one biological replicate (Intact, n = 2; d3, n = 3; d7, n = 2). Lines connect tissue group means. Statistical comparisons were performed using two-sided Wilcoxon test with Benjamini-Hochberg correction; ns = not significant.

(B) Stacked bar plots showing proportional distribution of annotated cell types in fractured and contralateral samples from marrow and cortex compartments at each timepoint.

(C) Volcano plots showing differential abundance analysis of cell types between fractured and contralateral sides in marrow and cortex. No significant differences were detected across timepoints in the absence of injury, supporting the use of contralateral tissue as an internal control.

Data are provided in Supplementary Data 3.

Suppl. Figure 4

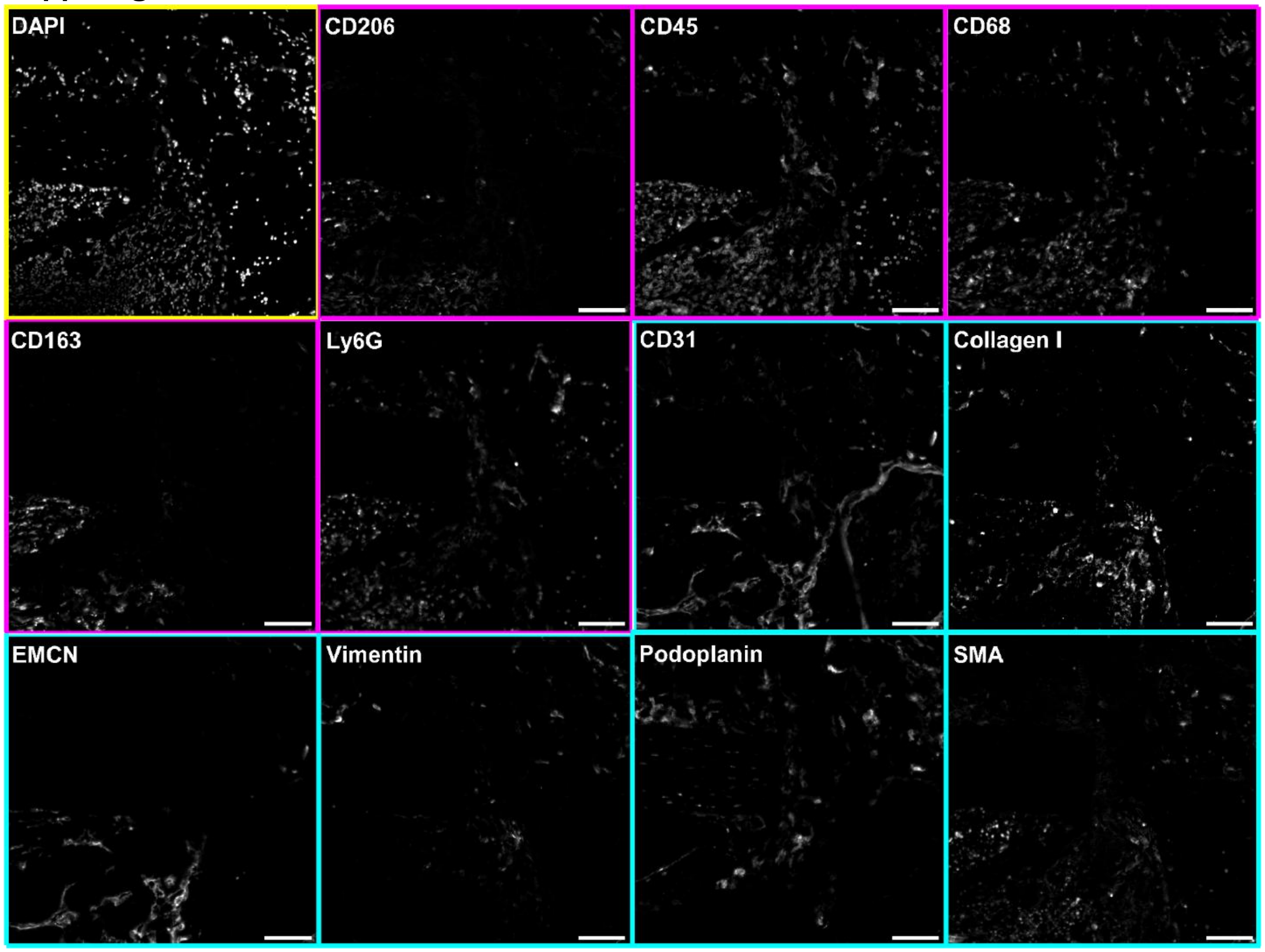

Suppl. Fig. 4: MELC panel for cortical bone tissue

Representative field of view from young bone tissue near the cortical surface, sequentially stained using a 12-marker MELC panel. Markers include immune cell populations (magenta), stromal and endothelial cell markers (cyan) and nuclei stainings (yellow). Each marker was visualized in the same tissue region through cyclic immunofluorescence. Image resolution is 2048x2048 pixels, corresponding to 665x665 µm. Scale bar: 100 µm.

Suppl. Figure 5

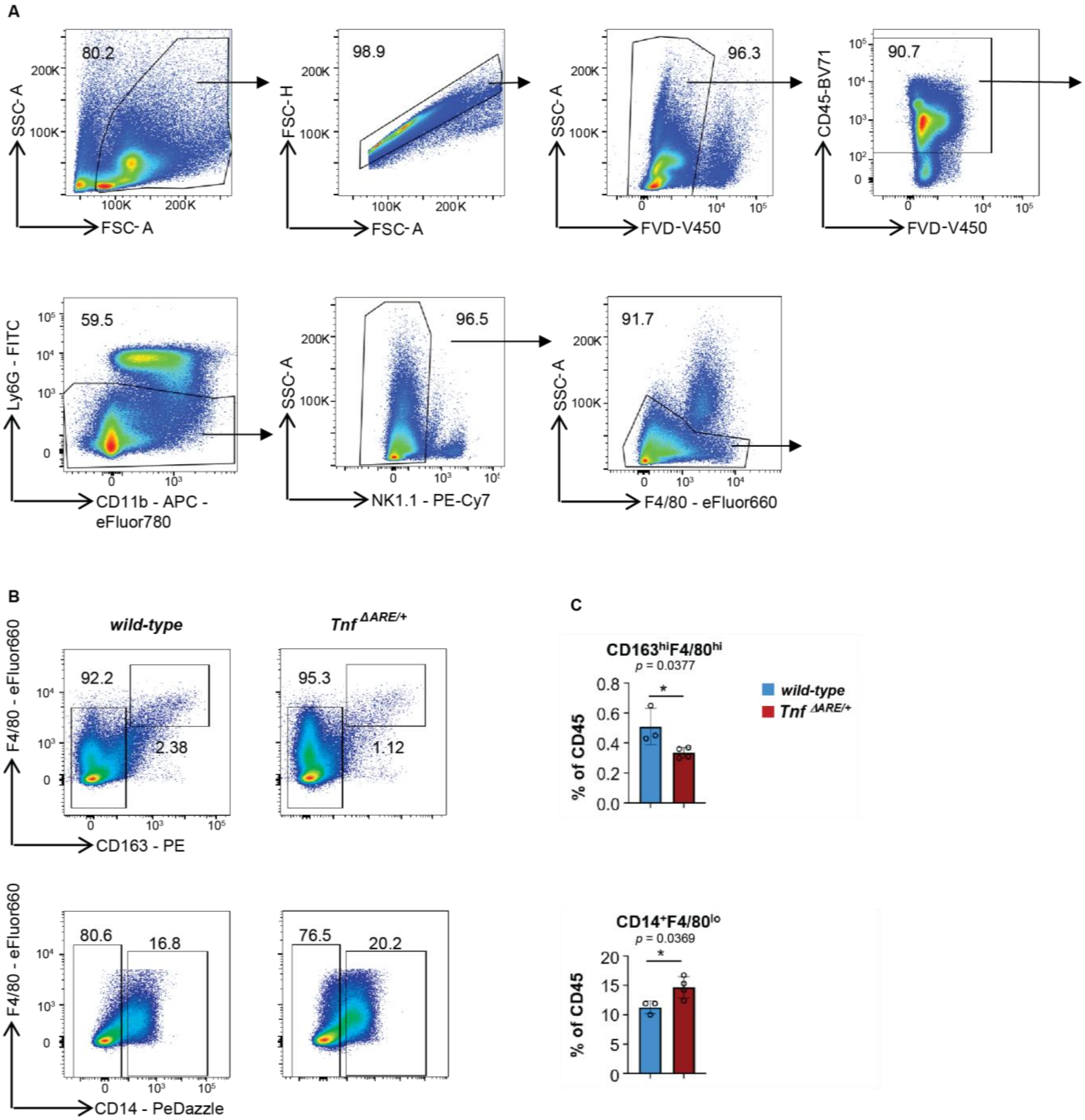

Suppl. Fig. 5:

Gating strategy and quantification of fracture-associated macrophage subsets (related to Figure 4F).

(A) Gating strategy used for the identification of fracture associated macrophage subsets from mouse bone marrow.

(B) Representative plots showing gating of $CD163^{+}F4/80^{hi}$ macrophages and $CD14^{+}F4/80^{lo}$ macrophage populations corresponding to $CD163^{hi}$ and fracture- associated *Spp1*^{hi} macrophages, based on their transcriptional analysis shown in Figure 4D.

(C) Quantification of gated populations as percentage of $CD45^{+}$ cells in wild-type (*Tnf*$^{+/+}$) and *Tnf*$^{\Delta ARE}$ conditions. Data points represent individual mice (n = 3).

Data are provided in Supplementary Data 4.

Suppl. Figure 6

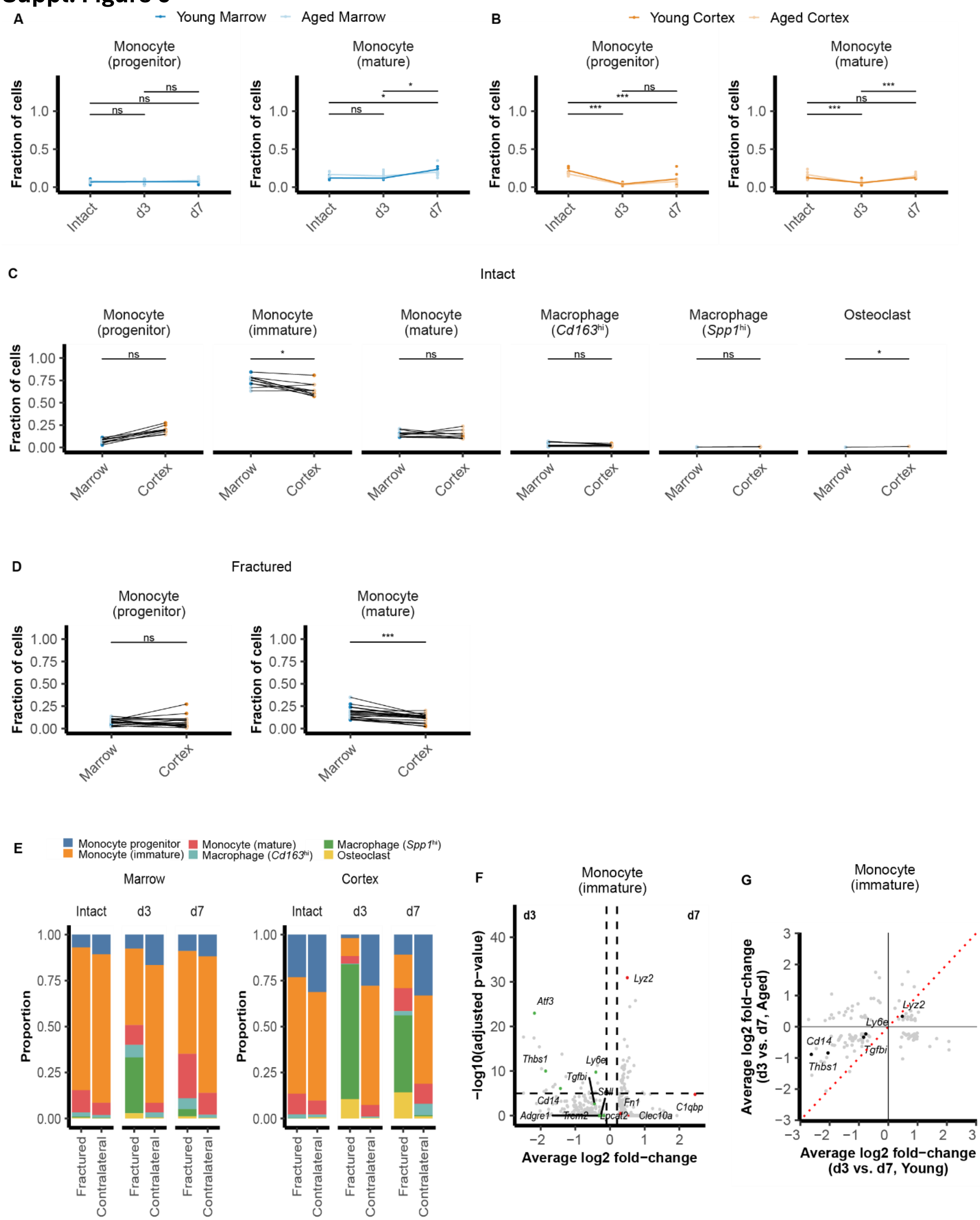

Suppl. Fig. 6: Distinct temporal and tissue-specific differentiation trajectories of monocyte-derived macrophages during early fracture healing.

(A-B) Fractional abundance of monocyte progenitors and mature monocytes across intact, day 3, and day 7 conditions in young (blue) and aged (orange) mice, showing distinct dynamics over time. Data points represent biological replicates (intact, n = 10; d3, n = 10; d7, n = 9), and lines depict group means. Comparisons across time points were performed using two-sided Wilcoxon rank-sum tests with Benjamini-Hochberg correction for multiple testing; * $P < 0.05$, ** $P < 0.01$, *** $P < 0.001$, **** $P < 0.0001$; ns = not significant. The exact *P* values are provided in the Suppl. Data file.

(C-D) Paired comparisons of monocyte and macrophage subtypes between marrow and cortex within replicates in (C) intact samples (n = 10) and (D) fractured samples (n = 19). Tissue-specific differences were assessed using paired one-sided Wilcoxon signed-rank tests; Intact, Monocyte (mature) $P = 0.048$ and Intact, Osteoclast $P = 0.034$; Fractured, Monocyte (mature) $P = 0.0002$; ns = not significant.

(E) Stacked bar plots showing proportional distribution of monocyte/macrophage subsets across marrow and cortex compartments at all timepoints, comparing fractured and contralateral sides.

(F) Volcano plot showing differentially expressed genes in immature monocytes at day 3 vs. day 7 post-osteotomy in young mice, highlighting changes associated with early activation and remodeling. Genes labeled in green indicate significant upregulation at d3; genes in red are upregulated at d7. Differential expression analysis was performed using two-sided Wilcoxon rank-sum tests with Benjamini-Hochberg adjustment for multiple testing.

(G) Comparative gene expression plot showing $\log_2$ fold-change in immature monocytes between day 3 and day 7 in young versus aged mice. Red dotted line indicates identity (no age effect). Highlighted genes indicate consistent regulation across age.

Data are provided in Supplementary Data 5.

Suppl. Figure 7

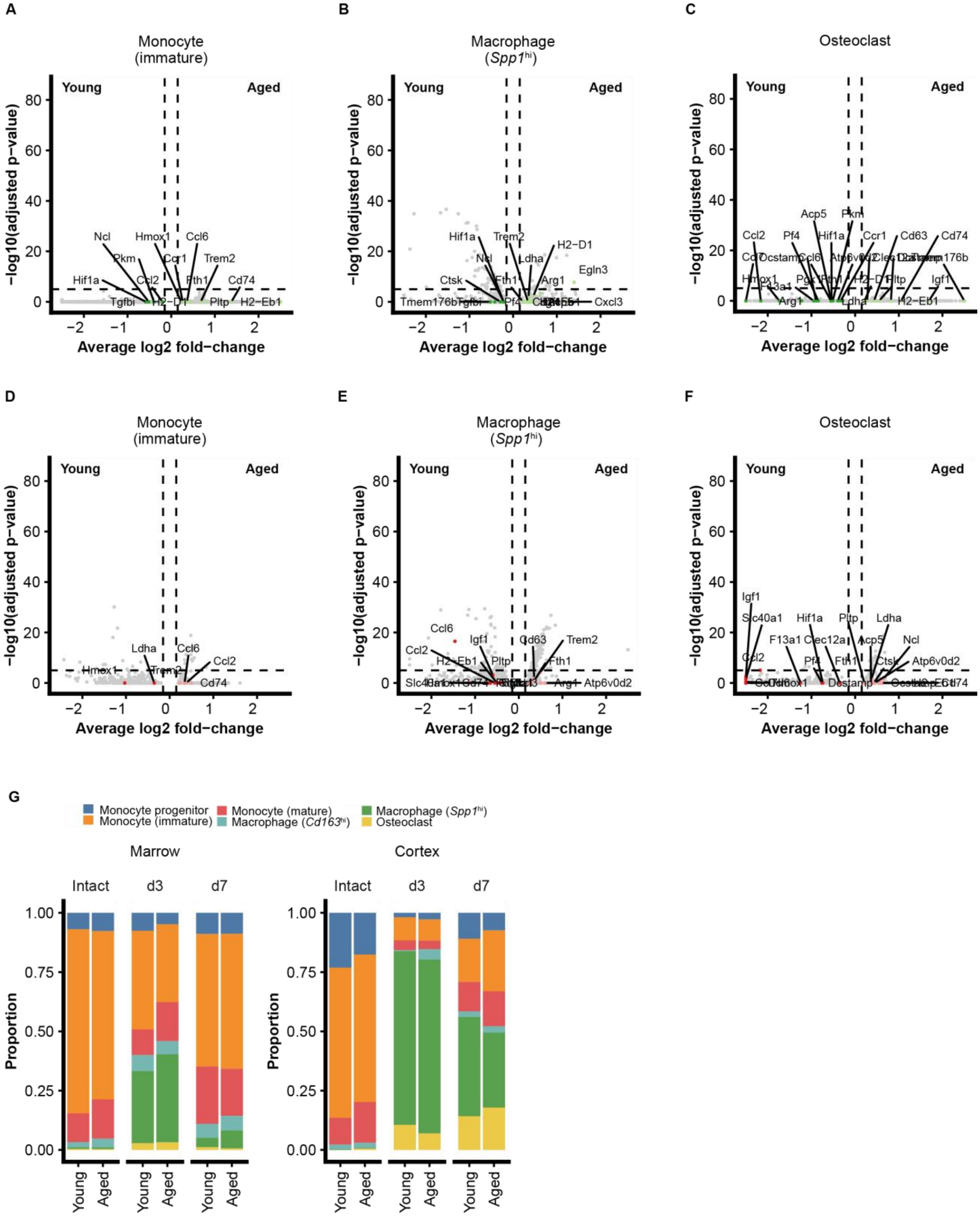

Suppl. Fig. 7: Age-dependent transcriptional changes in monocytes, *Spp1*^{hi} macrophages and osteoclasts across marrow and cortical niches during early fracture healing.

(A–C) Volcano plots showing differential gene expression between young and aged mice at day 3 post-osteotomy for (A) immature monocytes, (B) *Spp1*^{hi} macrophages, and (C) osteoclasts, highlighting age-associated shifts in inflammatory, metabolic, and osteoclastogenic programs. Differential expression analysis was performed using two-sided Wilcoxon rank-sum tests with Benjamini-Hochberg adjustment for multiple testing.

(D–F) Differential gene expression analysis at day 7 comparing young and aged mice for the same three populations, illustrating early age-dependent transcriptional rewiring prior to osteoclast emergence. Differential expression assessed using two-sided Wilcoxon test with Benjamini–Hochberg correction for multiple testing.

(G) Stacked bar plots depicting proportional changes in the myeloid subsets across marrow and cortex compartments in intact, day 3, and day 7 samples in young versus aged mice, showing niche- and time-specific alterations in lineage distribution.

Data are provided in Supplementary Data 6.

Suppl. Table S1: Antibody panel and acquisition settings used for MELC, including antibody sources, clones, fluorophores, dilutions, and imaging parameters.

	antibody information				pipetting information			MELC acquisition information			
cycle	marker	fluorophore	company	clone	well	dilution	PBS (µl)	antibody (µl)	incubation time	bleaching time	cleaning cycles
1	Blocking				E1		60.0		900	900	9
	Blocking									900	9
	Blocking									0	0
2	CD206	PE	Biolegend	C068C2	E2	50	55.2	2.4	2400	600	6
	SMA	FITC	Abcam	1A4		50		2.4		900	9
	DAPI		Roche			5000		0.0		0	0
3	EMCN	PE	Invitrogen	eBioV.7C7	E3	400	59.7	0.3	1800	900	9
4	Ly6G	PE	Biolegend	1A8	E4	100	58.8	1.2	1800	900	9
5	COL-I	PE	Novus Bio	polyclonal	E5	100	58.8	1.2	1800	900	9
6	CD163	PE	Invitrogen	TNKUP	E6	200	59.4	0.6	2700	900	9
7	FN	PE	Novus Bio	2755-8	E7	400	59.7	0.3	2700	900	9
8	CD68	PE	Miltenyi	REA835	E8	10	48.0	12.0	2400	900	9
9	CD45	PE	Invitrogen	30-F11	E9	300	59.0	0.4	1800	900	9
	Vimentin	A488	Abcam	EPR3776		200		0.6		900	9
10	PDPN	PE	Invitrogen	eBio8.1.1	E10	200	57.0	0.6	1800	0	0
	CD31	FITC	R&D systems	polyclonal		50		2.4		1200	12
11	Sytox		ThermoFisher		E11	400	59.7	0.3	900	0	0

Suppl. Table S2: Antibody panel used for flow cytometry, including fluorophores, suppliers, catalogue numbers, clone identifiers, lot numbers, and staining dilutions.

antibody information						pipetting information
Marker	Fluorophore	Company	Catalogue number	Lot number	Clone	dilution
CD45	BV711	BD Horizon	563709	3072697	30 -F11	1:400
Ly6G	FITC	Biolegend	127606	B435608	1A8	1:200
Ly6C	PerCP-eF710	Invitrogen	45-5932-92	1996378	HK1.4	1:400
CD163-PE	PE	Invitrogen	12-163-182	2896790	TKNUPJ	1:100
CD14	PEDazzle	Biolegend	123326	B398450	Sa14-2	1:100
Nk1.1	PeCy7	Invitrogen	25-5941-82	2686516	PK136	1:100
F4/80	eFluor660	Invitrogen	50-4801-82	2989386	BM8	1:100
CD11b	APC-eFluor780	Invitrogen	47-0112-82	2560618	M1/70	1:150
CD3e biotin		Invitrogen	13-0031-82	3053659	145-2C11	1:300
CD5 biotin		Invitrogen	13-0051-85	2355944	53-7.3	1:150
CD19 biotin		Invitrogen	13-0191-85	2433463	MB19.1	1:150
NKp46 biotin		Invitrogen	13-3351-82	2518332	29A1.4	1:100
siglecf biotin		Biolegend	155512	B488098	S17007L	1:150
Streptavidin V500		BD Horizon	561419	4015931		1:800
