# Supplementary material for: The cortical microenvironment drives early immune organization and controls early osteoclastogenesis in bone healing
Source: Nat Commun. 2026 Aug 27;17:9154. doi: 10.1038/s41467-026-77196-7 (PMC13522349; doi:10.1038/s41467-026-77196-7)
Supplement: Supplementary file 3 [file 41467_2026_77196_MOESM2_ESM.pdf]

nature portfolio

Peer Review File

The cortical microenvironment drives early immune organization and controls early osteoclastogenesis in bone healing

Corresponding Author: Professor Georg Duda

This file contains all reviewer reports in order by version, followed by all author rebuttals in order by version.

Version 0:

Reviewer comments:

Reviewer #1

(Remarks to the Author)
The strength of this study lies in attempting to clarify a fundamental but unresolved question in bone regeneration: which myeloid populations give rise to osteoclasts during the earliest phase of repair, and how this process is regulated by the tissue environment? The work establishes a conceptual framework in which spatially restricted immune–stromal coordination within the cortex is indispensable for timely osteoclast genesis, while also providing insight into how aging disrupts this finely tuned process. However, there are some key claims that needs additional data to support.

Major Revisions

A central claim of the manuscript is the identification of a distinct Spp1-high macrophage subset within the cortical niche that serves as a transitional precursor for osteoclasts. However, the support for this conclusion is based exclusively on transcriptomic inference (single-cell RNA-seq and spatial mapping). The experiments presented convincingly demonstrate that Spp1high macrophages are enriched at early timepoints and occur at higher frequency within the cortical microenvironment compared to the bone marrow, but these findings only establish their spatial and temporal association with osteoclastogenesis, not a direct precursor–product relationship. This limitation is particularly important given that prior studies have already implicated SPP1high macrophages in a wide range of regenerative and pathological contexts, including bone repair1, polytrauma2, vascular calcification3, and osteoclast–osteoblast signaling via SPP14. Broader perspectives further emphasize that SPP1high macrophages represent a conserved and disease-associated subset with therapeutic potential across tissues5,6. Because this body of literature already exists, the bar for novelty is higher, and future progress in the field requires moving beyond transcriptomic correlations toward direct experimental validation.

To substantiate the claim that cortical Spp1-high macrophages are bona fide osteoclast progenitors, functional validation is essential. While the current study establishes their early presence and enrichment in the cortical niche, it does not directly prove that these cells differentiate into osteoclasts. Such validation could include isolating Spp1high macrophages and testing their osteoclastogenic potential in vitro, employing fate-mapping approaches to trace their differentiation in vivo, or using targeted depletion strategies to assess their contribution to bone healing. Without these kinds of experimental data, the novelty of positioning Spp1high macrophages as direct osteoclast precursors is significantly weakened, and the manuscript risks overstating its conclusions.

Second, the authors conclude that immature monocytes, Spp1high macrophages, and osteoclasts exhibit “similar activation and differentiation patterns across age groups” and that their molecular programs remain intact. While the kinetic analyses (intact, d3, d7 Figure 4F, 4G) demonstrate population dynamics within each age group, they primarily assess changes over time rather than providing a direct comparison between young and aged mice at matched stages. Similarly, the DEG analysis suggests broadly conserved transcriptional programs (Figure 6c), but it does not fully address whether more subtle age-related differences in gene expression trajectories or niche interactions might underlie impaired osteoclastogenesis in aged animals. Given that Spp1high macrophages are proposed as osteoclast precursors and potential therapeutic targets, a more explicit cross-age analysis of both population abundance (e.g., young vs. aged at d3 and d7) and transcriptional programs is critical. Without this, the impact of aging on this key precursor population remains insufficiently characterized, leaving a gap in the mechanistic interpretation.

References
1. https://doi.org/10.1038/s41413-024-00347-3
2. https://doi.org/10.1038/s41413-025-00444-x
3. https://doi.org/10.1038/srep40253
4. https://doi.org/10.1016/j.biomaterials.2023.122367
5. https://doi.org/10.1096/fj.202403227R
6. https://doi.org/10.1111/imm.13952

Minor Revisions

1. Line 186–187: The cited in-text reference was not found and should be corrected or replaced.
2. Figure 3 (Panels A and B): The panels appear pixelated, making the line graphs difficult to interpret. Please improve the image resolution and consider increasing the line thickness for clarity.
3. Figure 5B: The use of red and green together is not color-blind friendly. Please adjust the color scheme to improve accessibility, if possible.
4. Figure 5E, F, and G: The age of the mice used for these experiments is not specified. Please clarify what age groups were included. More generally, for experiments where age is not variable, the manuscript should indicate the age of the mice used.

Reviewer #2

(Remarks to the Author)
I co-reviewed this manuscript with one of the reviewers who provided the listed reports. This is part of the Nature Communications initiative to facilitate training in peer review and to provide appropriate recognition for Early Career Researchers who co-review manuscripts.

Reviewer #3

(Remarks to the Author)
As someone who studies in the field of bone healing, immunology, and macrophage biology, I found the paper promising, particularly with its focus on the spatial relationship between macrophages and osteoclasts in primary bone healing. The authors employ advanced techniques like single-cell RNA sequencing and multi-epitope ligand cartography to offer valuable insights into cellular dynamics during cortical defect healing. This approach has the potential to significantly enhance our understanding of the cellular progression in bone healing.

However, the Introduction would benefit from a clearer explanation of how the model relates to traditional fracture healing. Specifically, does the model promote a typical healing response through endochondral ossification, or is it more consistent with primary bone healing? This distinction is critical for assessing the broader relevance of the findings.

Additionally, a more comprehensive review of the existing literature, particularly regarding myeloid lineage, macrophage subtypes, and their roles in osteoclastogenesis, would enhance the manuscript. This could be incorporated either in the introduction or data/analysis section, as it would help contextualize the results within the broader landscape of bone healing research.

In terms of validity, the methods were sound and appropriately applied, ensuring reliable results. However, in the Data & Methodology section, the inclusion of numerical data would greatly strengthen the paper. For example, providing specific population changes or a clearer definition of what constitutes a "substantial difference" in frequency would help convey the significance of these shifts over time in the context of bone healing.

Additionally, a more explicit discussion of statistical significance would improve clarity throughout the manuscript. At times, I found myself needing more concrete details and a better connection to early bone healing processes. Clearer distinctions between significant and non-significant results would help the reader better understand the impact of these findings.

Further, I suggest that the limitations of this model and its associated methodologies be more thoroughly discussed to provide a clearer picture of potential confounding factors or constraints in the findings.

In summary, while the study offers promising insights, refining the presentation of data and clarifying certain points would greatly improve its overall impact.

Version 2:

Reviewer comments:

Reviewer #1

(Remarks to the Author)

The authors have adequately answered this reviewer's questions.

Open Access This Peer Review File is licensed under a Creative Commons Attribution 4.0 International License, which permits use, sharing, adaptation, distribution and reproduction in any medium or format, as long as you give appropriate credit to the original author(s) and the source, provide a link to the Creative Commons license, and indicate if changes were made.
In cases where reviewers are anonymous, credit should be given to 'Anonymous Referee' and the source.
The images or other third party material in this Peer Review File are included in the article’s Creative Commons license, unless indicated otherwise in a credit line to the material. If material is not included in the article’s Creative Commons license and your intended use is not permitted by statutory regulation or exceeds the permitted use, you will need to obtain permission directly from the copyright holder.
To view a copy of this license, visit https://creativecommons.org/licenses/by/4.0/

We thank the reviewers for the comprehensive and helpful feedback. We have addressed all issues raised and reply here in a point-by-point manner to each aspect. We would like to express our gratitude and hope that with the implementation of these changes, the manuscript now fulfills the requirements for publication.

Reviewer #1

We would like to thank reviewer #1 for critically reviewing our paper. Below you can find a point-by-point reply.

Reviewer's comment: A central claim of the manuscript is the identification of a distinct Spp1-high macrophage subset within the cortical niche that serves as a transitional precursor for osteoclasts. However, the support for this conclusion is based exclusively on transcriptomic inference (single-cell RNA-seq and spatial mapping). The experiments presented convincingly demonstrate that $Spp1^{high}$ macrophages are enriched at early timepoints and occur at higher frequency within the cortical microenvironment compared to the bone marrow, but these findings only establish their spatial and temporal association with osteoclastogenesis, not a direct precursor–product relationship. This limitation is particularly important given that prior studies have already implicated SPP1high macrophages in a wide range of regenerative and pathological contexts, including bone repair[1], polytrauma[2], vascular calcification[3], and osteoclast–osteoblast signaling via SPP1[4]. Broader perspectives further emphasize that SPP1high macrophages represent a conserved and disease-associated subset with therapeutic potential across tissues[5,6]. Because this body of literature already exists, the bar for novelty is higher, and future progress in the field requires moving beyond transcriptomic correlations toward direct experimental validation.

To substantiate the claim that cortical Spp1-high macrophages are bona fide osteoclast progenitors, functional validation is essential. While the current study establishes their early presence and enrichment in the cortical niche, it does not directly prove that these cells differentiate into osteoclasts. Such validation could include isolating Spp1high macrophages and testing their osteoclastogenic potential in vitro, employing fate-mapping approaches to trace their differentiation in vivo, or using targeted depletion strategies to assess their contribution to bone healing. Without these kinds of experimental data, the novelty of positioning Spp1high macrophages as direct osteoclast precursors is significantly weakened, and the manuscript risks overstating its conclusions.

Response: We thank the reviewer for these important and related comments. We agree that positioning Spp1hi macrophages as osteoclast-associated precursors requires both appropriate contextualization within the existing literature and functional validation beyond transcriptomic inference.

We acknowledge that similar $Spp1^{hi}$ macrophage phenotypes have been previously described in other regenerative and pathological contexts; however, while these prior descriptions of this phenotype have provided important functional and descriptive insights into Spp1/OPN-associated myeloid states, they have not established a spatially resolved framework that defines how this phenotype emerges, differentiates and behaves during regeneration, especially fracture healing. In addition to describing key transcriptional features that are associated with this phenotype in the context of fracture healing, our manuscript generates substantial, additional insights into the emergence and differentiation trajectory of these macrophages in fracture healing. For example, by conducting pseudotemporal ordering of myeloid cells in our dataset, we establish a trajectory in which myeloid precursors in the bone marrow differentiate into $Spp1^{hi}$ macrophages during fracture healing. In addition, this trajectory suggests that fracture-associated $Spp1^{hi}$ macrophages likely have the potential to further differentiate into dual cell fates, including pro-regenerative $Cd163^{hi}$ macrophages as

well as osteoclasts. In addition, as noted by the reviewer, our manuscript provides, for the first time, evidence for the association of fracture-associated *Spp1*^{hi} macrophages with the cortex during fracture repair. To our knowledge, similar findings regarding the differentiation of this phenotype and their spatial positioning have not been clearly established in other regenerating organs. Thus, the prior description of *Spp1*^{hi} macrophages in other contexts does not undermine the novelty of this manuscript; rather, by establishing important findings regarding the emergence and behavior of this macrophage phenotype in bone healing, this manuscript advances knowledge about the behavior, differentiation, and function of this important phenotype and introduces the notion that *Spp1*^{hi} macrophages may be an important source of osteoclasts in fractured bone sites.
In the revised document, we therefore explicitly clarify what is unique about our observations in our fracture healing model, including:

- The differentiation of this phenotype from monocyte precursors
- The rapid emergence of this phenotypic during early fracture healing,
- Their selective enrichment of this phenotype to the cortical niche
- The role of this phenotype as a branching point between macrophage and osteoclast fates
- The transcriptional alignment of this phenotype with osteoclast programs,
- The spatial co-localization of this phenotype with early osteoclasts
- The conservation of this phenotype's behavior in aged animals.

To emphasize these findings, we have made the following revisions to our manuscript (additions highlighted in bold):

Line 533 (Discussion): *"To address this discrepancy, we next aimed to identify transcriptional states that may represent osteoclast precursors during early healing.* ***Consistent with reports in other injured*** *tissues, such as the muscle[42], lung[58], kidney[59] and even bone****, our analysis also revealed a distinct Spp1^{hi} macrophage population. In the unique context of fracture healing, we were able to resolve features of the fracture-associated Spp1^{hi} macrophage state that have not been defined previously in bone: its differentiation trajectory, dynamic emergence during early timepoints in the healing process, enrichment at the cortex, and its position at the transition toward osteoclast identity. Trajectory analysis demonstrated that fracture-associated Spp1hi macrophages form a key intermediate between Ly6C^{hi} monocytes, reparative CD163^{hi} macrophages and osteoclasts. Their mixed M1/M2-like transcriptional profile indicates responsiveness to local cues produced in the cortical microenvironment. Their upregulation of osteoclast-associated transcripts is in agreement with their capacity to form osteoclasts ex vivo. The accumulation of Spp1^{hi} macrophages at the cortex, coincident with rising osteoclast numbers, suggests that their differentiation is spatially instructed by the bone healing microenvironment rather than intrinsic. Notably, this transitional state was preserved in aged mice, even though downstream osteoclastogenic output was diminished, suggesting that age primarily alters the niche rather than the precursor state itself."***

Importantly, in response to the reviewer's concern, we have now complemented our transcriptomic analyses with functional validation. We performed ex vivo osteoclastogenesis assays from macrophage subsets isolated by flow cytometry and cell sorting. These assays functionally assess osteoclast-forming capacity across myeloid populations aligned with the transcriptional states identified in our single-cell analysis. Because *Spp1*^{hi} macrophages cannot be directly isolated based on Spp1 protein expression, we used surface marker combinations derived from our single-cell dataset to enrich for populations corresponding to this state along the inferred trajectory.

Specifically, we isolated transitional Spp1^{hi} macrophages (identified as CD14^{+} F4/80^{lo}) and more mature Cd163^{hi} macrophages (F4/80^{hi}). Upon M-CSF and RANKL stimulation, the *Spp1*^{hi}-aligned population generated significantly more large, multinucleated TRAP-positive osteoclasts than Cd163^{hi} macrophages (Figure 4F), demonstrating that osteoclastogenic capacity peaks at this transitional stage. We further show that this property is preserved under chronic inflammatory conditions (*Tnf*$^{\Delta ARE}$), where increased osteoclastogenesis is driven by expansion of precursor abundance rather than altered per-cell differentiation potential.

To explicitly reflect these additional analyses, we added the following sentence to the Results:

> Line 296 (Results): ***"To functionally assess osteoclast differentiation capacity along the inferred myeloid trajectory, we performed osteoclastogenesis assays where we plated equal numbers of cells using flow-sorted myeloid populations. Based on marker expression patterns identified in our single-cell dataset, we isolated two phenotypically distinct populations representing successive stages along the trajectory: transitional Spp1^{hi} macrophages (identified as CD14^{+} F4/80^{lo}) and Cd163^{hi} macrophages (identified as CD163hiF4/80hi) (Fig. 4D and Suppl. Figure 5A-B). Upon stimulation with M-CSF and RANKL, Spp1^{hi} macrophages generated large, multinucleated TRAP positive osteoclasts, demonstrating their osteoclast differentiation potential (Figure 4F). In contrast, Cd163^{hi} macrophages formed very few osteoclasts. These findings indicate that Spp1^{hi} macrophages have a higher capacity to form osteoclasts, compared to other fracture-associated macrophage states, consistent with the notion that they represent a transitional macrophage state, which in the context of bone healing gives rise to fracture-associated osteoclasts.***
>
> ***To determine whether inflammatory conditions result in increased representation of Spp1^{hi} macrophages and/or increased osteoclast differentiation capacity, we performed these assays using macrophage subsets isolated from wild-type (Tnf$^{+/+}$) and Tnf$^{\Delta ARE/+}$ mice. In the latter, increased expression of TNF recapitulates chronic TNF driven inflammation associated with enhanced osteoclastogenesis and pathological bone loss. Chronic TNF-driven inflammation did not significantly affect the relative osteoclastogenic efficiency of the examined subsets (Figure 4F). Instead, inflammatory conditions primarily increased the abundance of osteoclast-competent precursor cells, without altering their per-cell differentiation potential (Suppl. Figure 5C). Together, these findings support a model in which the representation of Spp1^{hi} macrophages with high osteoclastogenic capacity is increased under inflammatory conditions, while at the same time the representation of Cd163hi macrophages, which have a lower capacity to form osteoclasts, is decreased."***

Importantly, we have **revised the manuscript to more precisely reflect the strength and limitations of our conclusions**. We now position fracture-associated Spp1^{hi} macrophages as a *transitional, osteoclast-aligned state with heightened osteoclastogenic potential*, rather than definitively proven direct precursors. This refinement is reflected in the Abstract and Discussion, where we explicitly acknowledge that a precursor–product relationship remains to be formally established and will require future lineage-tracing or depletion studies:

> Line 47 (Abstract): "*We identified a Spp1^{hi} macrophage subset restricted to the cortex,* ***which represented a transitional monocyte-derived state that transcriptionally aligned with early osteoclast differentiation*****.**"
>
> Line 54 (Abstract): "*Together, our findings identify* ***a distinct macrophage state likely upstream of osteoclast differentiation*** *and reveal early, cortex-specific niche activity supporting osteoclastogenesis*."

Line 585 (Discussion): *"Several limitations of this work should be acknowledged.* ***First, although our data position Spp1^{hi} macrophages upstream of osteoclasts in transcriptional space and spatially associate them with early osteoclasts, we cannot establish a direct precursor-product relationship.*** *Functional lineage tracing or targeted depletion assays will be required to determine whether Spp1^{hi} macrophages directly contribute to the osteoclast pool during fracture healing. Second, our single-cell RNA sequencing relied on tissue dissociation and may therefore underrepresent fragile or spatially embedded cells. Finally, although our analyses highlight age-related changes in niche activity rather than intrinsic cellular defects, additional work integrating functional assays or in situ perturbations will be needed to better define how aging alters the coordination between immune and stromal compartments during early fracture healing."*

Reviewer's comment: The authors conclude that immature monocytes, Spp1high macrophages, and osteoclasts exhibit "similar activation and differentiation patterns across age groups" and that their molecular programs remain intact. While the kinetic analyses (intact, d3, d7 Figure 4F, 4G) demonstrate population dynamics within each age group, they primarily assess changes over time rather than providing a direct comparison between young and aged mice at matched stages. Similarly, the DEG analysis suggests broadly conserved transcriptional programs (Figure 6c), but it does not fully address whether more subtle age-related differences in gene expression trajectories or niche interactions might underlie impaired osteoclastogenesis in aged animals. Given that Spp1high macrophages are proposed as osteoclast precursors and potential therapeutic targets, a more explicit cross-age analysis of both population abundance (e.g., young vs. aged at d3 and d7) and transcriptional programs is critical. Without this, the impact of aging on this key precursor population remains insufficiently characterized, leaving a gap in the mechanistic interpretation.

Response: We thank the reviewer for this insightful suggestion and agree that a more direct cross-age comparison would help clarify the impact of aging on the myeloid-osteoclast lineage. In response, we performed additional cross-age differential expression analyses and compiled them into a new Supplementary Figure (Suppl. Fig. 7).

To test whether molecular programs are preserved across age, we performed pseudobulk differential expression comparing immature monocytes, *Spp1*^{hi} macrophages and osteoclasts between young and aged mice at day 3 and day 7 post-osteotomy. Across all three monocyte-derived populations and both timepoints, we observed only minimal transcriptional differences between young and aged mice. This suggests that (1) the overall osteoclastogenic transcriptional program is preserved with age, (2) age does not substantially alter monocyte-to-osteoclast differentiation states and (3) age-related differences observed in osteoclast activity with the TRAP staining likely arise from niche-level changes.

To explicitly reflect these additional analyses, we added the following sentence to the Results:

> Line 407 : "*Crucially, these transcriptional programs were largely preserved in aged mice (Figure 6C, F, Suppl. Figure 6F-G and* ***Suppl. Figure 7). Direct cross-age pseudobulk comparisons at matched timepoints revealed only minimal transcriptional differences between young and aged mice in immature monocytes, Spp1^{hi} macrophages, and osteoclasts. The*** *DEG comparisons further revealed that the myeloid cells followed similar activation and differentiation patterns across age groups. While the abundance of these populations may differ, their molecular programs remained intact. This suggests that age-related impairments in osteoclastogenesis are more likely due to altered microenvironmental signals rather than intrinsic cellular dysfunction*."

Minor Revisions

1) **Line 186–187: The cited in-text reference was not found and should be corrected or replaced.** We thank the reviewer for noticing this. We have updated the reference and should now be listed accordingly.
2) **Figure 3 (Panels A and B): The panels appear pixelated, making the line graphs difficult to interpret. Please improve the image resolution and consider increasing the line thickness for clarity.** We have replaced Figure 3A-B panels with higher resolution.
3) **Figure 5B: The use of red and green together is not color-blind friendly. Please adjust the color scheme to improve accessibility, if possible.** To improve the accessibility of this figure, we added distinct point shapes (triangles for day 3 and squares for day 7).
4) **Figure 5E, F, and G: The age of the mice used for these experiments is not specified. Please clarify what age groups were included. More generally, for experiments where age is not variable, the manuscript should indicate the age of the mice used.** We thank the reviewer for pointing this out. The figure legend for Figure 5E-G and the corresponding section in the Methods (line 705) now specify the ages of the animals used. For these experiments, all mice were young (12-weeks), unless stated otherwise. We also reviewed the full manuscript to ensure that the age of the animals is explicitly indicated in each experimental description where relevant.

Reviewer #2

We thank reviewer #2 for their contribution to the evaluation of our manuscript and for their constructive engagement in the review process.

Reviewer #3

We would like to thank reviewer #3 for their constructive and thoughtful evaluation of our manuscript. Below you can find our detailed responses to the reviewers comments.

Reviewer's comment: The Introduction would benefit from a clearer explanation of how the model relates to traditional fracture healing. Specifically, does the model promote a typical healing response through endochondral ossification, or is it more consistent with primary bone healing? This distinction is critical for assessing the broader relevance of the findings.

Response: We thank the reviewer for raising this important point. The model used in our study is a stabilized femoral osteotomy, which recapitulates the physiological sequence of secondary (endochondral) bone healing, as previously described by Bonnarens & Einhorn (1984) and Bucher *et al.* (2019). In this model, bone regeneration proceeds through a well-defined series of stages: inflammation, formation of a soft and subsequently mineralized callus, and endochondral ossification, followed by remodeling of the newly formed bone. We have clarified this description in the introduction.

> Line 64 (additions highlighted in bold): *"Bone* ***regeneration in long bones like the femur typically proceeds through secondary healing. Following fracture, the area is rapidly filled with a hematoma, initiating the inflammatory phase and creating a provisional matrix shaped by coagulation and platelet activation[7]. During these early stages, the hematoma and adjacent bone compartments are infiltrated by*** *immune cells[8,9], such as neutrophils[10,11] and macrophages[12,13],* ***which orchestrate the transition from acute inflammation to tissue repair."***

To ensure that this context is also clear in the Discussion, we have adjusted the opening of the second paragraph to state that our analyses capture events within the early inflammatory phase of secondary fracture healing, during which the cortical region is a key site of initial immune activation as follows:

Line 481: *"By combining single-cell RNA sequencing with spatial imaging, we reveal that* ***within the early inflammatory phase of secondary fracture healing****, the cortical niche serves as the primary site of innate immune activation, tissue remodeling, macrophage clearing of tissue debris and osteoclastogenesis, while the bone marrow retains its role as a hematopoietic reservoir[2]."*

Reviewer's comment: A more comprehensive review of the existing literature, particularly regarding myeloid lineage, macrophage subtypes, and their roles in osteoclastogenesis, would enhance the manuscript. This could be incorporated either in the introduction or data/analysis section, as it would help contextualize the results within the broader landscape of bone healing research.

Response: We agree and have expanded the Introduction to include a concise overview of macrophage heterogeneity and its relevance to osteoclast formation and bone regeneration, citing key studies where possible:

Line 74: ***"Macrophages have historically been described along an M1/M2 axis, yet single-cell transcriptomics has revealed a far broader landscape of context-dependent phenotypes[16–20]. Among these, Spp1^{hi} macrophages represent a transcriptionally distinct and recurrently observed state across multiple tissues and inflammatory settings[19,21,22].*** *Within the bone environment, this functional diversity is further expanded by the presence of both monocyte-derived macrophages and long-lived tissue-resident populations such as osteal macrophages (osteomacs), which support osteoblast function and matrix homeostasis[23,24]. Their plasticity and responsiveness to local cues position macrophages as central regulators of the early* ***fracture*** *environment.*

*In addition to their regulatory roles****, myeloid cells also give rise to osteoclasts, the multinucleated cells specialized in resorbing mineralized bones. Multiple myeloid subsets, including circulating Ly6C^{hi} monocytes[25], dendritic cell-like precursors[26] and tissue-resident macrophages[25], can differentiate into osteoclasts under stimulatory conditions. Much of this insight comes from in vitro differentiation systems in which myeloid cells are exposed to*** *macrophage colony-stimulating factor (M-CSF; Csf1) and receptor activator of nuclear factor κB ligand (RANKL)[27,28]****, which have been instrumental for defining the molecular pathways of osteoclastogenesis."***

To extend this contextual framework to our data, we also included the following sentence in the Results:

Line 287: "*Interestingly, the fracture-associated Spp1hi macrophages emerged as the key bifurcation point, marking a potential decision node between a reparative macrophage identity and an osteoclast fate.* ***This observation extends previous models in which osteoclasts are thought to arise from multiple myeloid subsets, including monocytes and tissue-resident macrophages[25], by suggesting that an additional intermediate state may be involved during fracture healing.*** *Collectively, these data provide evidence that fracture-associated Spp1hi macrophages represent a transitional macrophage state which in the context of bone healing has the potential to differentiate to osteoclasts at the fracture site."*

Reviewer's comment: In terms of validity, the methods were sound and appropriately applied, ensuring reliable results. However, in the Data & Methodology section, the inclusion of numerical data would greatly strengthen the paper. For example, providing specific population changes or a clearer definition of what constitutes a "substantial difference" in frequency would help convey the significance of these shifts over time in the context of bone healing.

Additionally, a more explicit discussion of statistical significance would improve clarity throughout the manuscript. At times, I found myself needing more concrete details and a better connection to early bone healing processes. Clearer distinctions between significant and non-significant results would help the reader better understand the impact of these findings.

Response: To improve the clarity of our manuscript, we have added fold-changes for all mentioned trends within myeloid and osteoclast populations when discussed in Figures 3-6. Specifically, we replaced qualitative descriptions with quantitative statements and added statistical results (p-values or effect sizes) wherever relevant to clarify the magnitude and significance of these changes. We have also carefully reviewed all figures and legends to ensure that statistical tests, p-values and sample sizes (n) are clearly reported. Non-significant comparisons are now explicitly labeled as 'ns'.

> For example: Line 184: "*Following the identification of major cell populations across compartments, we next quantified the relative proportions of each cell type to systematically characterize how fracture injury reshapes the cellular landscape over time. In the marrow, progenitor populations seemed to have one of the most dynamics changes (Suppl. Figure 2). Neutrophil-1, likely resembling neutrophil progenitors (Suppl. Figure 1), and monocyte progenitors expanded significantly* ***(Benjamini-Hochberg-adjusted p < 0.001)*** *at day 3. Notably, these expansions were not sustained as the progenitor levels returned to baseline level at day 7. In addition, mature immune cells also reacted to the osteotomy (Figure 3A). For instance, macrophages showed an* ***~10-fold*** *increase at day 3 followed by a similar decline at day 7, while dendritic cells and B cells showed the opposite (Suppl. Figure 2A). These shifts are consistent with an emergency hematopoietic response to recruit innate effectors from the marrow at day 3, and afterwards an inflammatory resolution phase by day 7. At the cortex, the temporal analyses were characterized by an accumulation of innate immune cells (Figure 3B). Macrophages also displayed here an* ***~30-fold*** *increase at day 3 and a decline at day 7. Furthermore, the Neutrophil-3 cluster expanded* ***almost 2-fold*** *over time, reaching their peak at day 7. In contrast to the marrow, the cortex had more cell types, such as dendritic cells and B cells but also plasma cells and erythrocytes, declining in levels during our chosen timepoints (Suppl. Figure 2B). This likely reflected not a loss of these cell types at the cortex, but rather a proportional shift for innate immunity at the cortex after osteotomy*."

Reviewer's comment (summary): Further, I suggest that the limitations of this model and its associated methodologies be more thoroughly discussed to provide a clearer picture of potential confounding factors or constraints in the findings.

Response: We appreciate this suggestion and have expanded the Discussion (lines 585-595) to outline the main limitations of our approach:

> ***"Several limitations of this work should be acknowledged. First, although our data position fracture-associated Spp1^{hi} macrophages upstream of osteoclasts in transcriptional space and spatially associate them with early osteoclasts, we cannot yet establish a direct precursor-product relationship. Lineage tracing using fate-mapping or targeted depletion will be required to determine whether Spp1^{hi} macrophages directly contribute to the osteoclast pool during fracture healing. Second, our single-cell RNA sequencing relied on tissue dissociation and may therefore underrepresent fragile or spatially embedded cells. Finally, although our***

analyses highlight age-related changes in niche activity rather than intrinsic cellular defects, additional work integrating functional assays or in situ perturbations will be needed to better define how aging alters the coordination between immune and stromal compartments during early fracture healing.”
