# Supplementary material for: The cortical microenvironment drives early immune organization and controls early osteoclastogenesis in bone healing
Source: Nat Commun. 2026 Aug 27;17:9154. doi: 10.1038/s41467-026-77196-7 (PMC13522349; doi:10.1038/s41467-026-77196-7)
Supplement: Supplementary file 4 [file 41467_2026_77196_MOESM3_ESM.pdf]

File Name: Supplementary Data 1
Description: Statistical source data for the analyses presented in Suppl. Figure 1.

File Name: Supplementary Data 2
Description: Statistical source data for the analyses presented in Suppl. Figure 2.

File Name: Supplementary Data 3
Description: Statistical source data for the analyses presented in Suppl. Figure 3.

File Name: Supplementary Data 4
Description: Statistical source data for the analyses presented in Suppl. Figure 5.

File Name: Supplementary Data 5
Description: Statistical source data for the analyses presented in Suppl. Figure 6.

File Name: Supplementary Data 6
Description: Statistical source data for the analyses presented in Suppl. Figure 7.
